# Rodent malaria parasites detected in the invasive *Rattus rattus* in Gabon

**DOI:** 10.1016/j.ijppaw.2025.101112

**Published:** 2025-07-05

**Authors:** Clark Mbou-Boutambe, Larson Boundenga, Fanny Degrugillier, Philippe Gauthier, Céline Arnathau, Ana Rivero, Laurent Granjon, Virginie Rougeron, Franck Prugnolle

**Affiliations:** a Unité de Recherche en Écologie de La Santé (URES), Centre Interdisciplinaire de Recherches Médicales de Franceville (CIRMF), Franceville, BP 769, Gabon; b École Doctorale Régionale D'Afrique Centrale en Infectiologie Tropicale (EDR), Franceville, BP 876, Gabon; c Département D'Anthropologie, Université de Durham, South Road, Durham DH1 3LE, UK; d Institut de Recherche pour le Développement (IRD), Maladies Infectieuses et Vecteurs, Écologie, Génétique, Évolution et Contrôle (MIVEGEC) (Université de Montpellier-IRD 224-CNRS 5290), Montpellier 34394, UK; e France CBGP, IRD, CIRAD, INRAE, Institut Agro, Université de Montpellier, Montpellier, France; f International Research Laboratory-REHABS, CNRS-Nelson Mandela University, Nelson Mandela University George Campus, George 6531, South Africa; g Sustainability Research Unit, Nelson Mandela University, George Campus, Madiba Drive, 6529 George, South Africa

**Keywords:** Black rat, *Plasmodium yoelii*, Invasive species, Rural areas, Gabon

## Abstract

Invasive species are increasingly recognized for their role in reshaping host–parasite dynamics. This study reports the first molecular detection of *Plasmodium yoelii* in the invasive black rat (*Rattus rattus*) in Gabon, based on a systematic molecular screening of 527 rodents captured in rural villages between 2021 and 2022. Two *R. rattus* individuals tested positive for *P. yoelii*, with phylogenetic analysis confirming identity with strains previously isolated from native rodents in the region. These findings challenge the traditional view that rodent malaria parasites are restricted to native hosts and highlight *R. rattus* as a potential, albeit likely incidental, host within local *Plasmodium* transmission networks. Despite a low infection prevalence (0.38 %), this result raises important questions about the capacity of invasive rodents to integrate into local parasite cycles and influence disease dynamics.

## Highlights

• Natural infection of *Rattus rattus* by rodent *Plasmodium*.
• First detection of *Plasmodium yoelii* in invasive *Rattus rattus* in Africa.
• *Rattus rattus* may act as an incidental reservoir of rodent malaria.

## Graphical abstract

**Figure:**
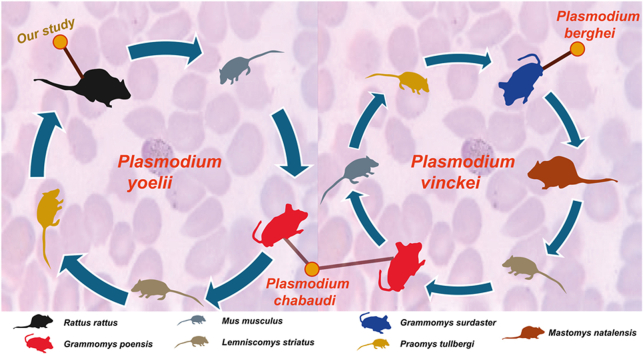

## 1 Introduction

Invasive species have been shown to have a significant impact on the transmission of pathogens, often acting as novel reservoirs that reshape host-parasite interactions and alter disease transmission dynamics (Roy et al., 2023; Dunn and Hatcher, 2015). Their uncontrolled introduction into new ecosystems can facilitate the spillover of new parasites or disrupt existing transmission cycles (Morand et al., 2015a; Diagne et al., 2016). This is of special concern in areas where invasive species come into close contact with native hosts, potentially leading to changes in parasite prevalence or the emergence of novel diseases.

*Rattus rattus*, commonly known as the black rat, is one of the most widespread invasive rodents globally, thriving in diverse environments, including urban, rural, and forested habitats (Aplin et al., 2011; Feng and Himsworth, 2014; Happold, 2013; Mangombi et al., 2016). Due to its adaptability and proximity to human settlements, *R. rattus* is a well-documented carrier of multiple zoonotic pathogens, such as *Leptospira* (Houéménou et al., 2019) *Salmonella* (Falay et al., 2022), and hantaviruses (Filippone et al., 2016). The role of invasive species like *R. rattus* in the ecology of zoonotic diseases, particularly in tropical and subtropical regions, has become a critical focus of research, as these rodents often bridge the gap between wild and domestic environments (Morand et al., 2015b; Banks and Hughes, 2012).

In some cases, invasive species can act as reservoirs or amplifiers of native parasites, thereby increasing the risk of infection for local hosts and disrupting epidemiological dynamics (Chinchio et al., 2020). Once established in a new environment, these species can acquire endemic parasites and contribute to their spread, leading to an increase in parasite prevalence or shifts in infection patterns. This process may result in heightened transmission of these indigenous pathogens to native hosts (a phenomenon known as "spillback") or to other local species, thereby exacerbating parasite dynamics within the ecosystem (Kelly et al., 2009). A striking example comes from Christmas Island (Australia), where *Rattus rattus* has been found to harbor a high prevalence and diversity of helminths. This suggests that this invasive species could play a central role in maintaining and circulating local parasites, with potential consequences for native wildlife and human health (Dybing et al., 2018). To fully understand the ecological and health implications of parasite acquisition by invasive hosts, it is essential to investigate these interactions in biodiversity hotspots, where exotic and native species coexist and where pathogen diversity is high. Gabon, located in Central Africa, provides an ideal setting for such studies.

Gabon, a country renowned for its remarkable biodiversity, has been identified as a potential hotspot for zoonotic disease transmission, including malaria (Boundenga et al., 2016, 2017; Lendongo Wombo et al., 2023; Sima-Biyang et al., 2024). *Plasmodium* parasites, the causative agents of malaria, are known to infect a wide range of vertebrate hosts, including birds, reptiles, and mammals (Boundenga et al., 2016, 2017). In murine rodents, four *Plasmodium* species: *Plasmodium berghei*, *P. chabaudi*, *P. vinckei*, and *P. yoelii*—have been identified, each comprising multiple subspecies, none of which are infectious to humans (Landau and Chabaud, 1994; Perkins et al., 2007; Boundenga et al., 2019). Originally, these parasites were thought to be confined to a limited number of native rodent species endemic to the Congo Basin (Culleton, 2005; Killick-Kendrick, 1978; Landau and Chabaud, 1994). This was especially true for *P. yoelii*, *P. chabaudi*, and *P. vinckei*, which were believed to exclusively infect *Grammomys poensis* — formerly known as *Thamnomys rutilans*, the thicket rat (Landau and Chabaud, 1994; Perkins et al., 2007; Stephens et al., 2012). However, recent findings have challenged this assumption, revealing that *Plasmodium* parasites in rodents may have a broader host range than previously recognized and that host switching events may occur more frequently than expected (Boundenga et al., 2019).

*Plasmodium* parasites infecting rodents are primarily transmitted by blood-feeding mosquitoes, particularly those of the genus *Anopheles*. The host specificity of these parasites depends not only on the rodent species but also on the vectors capable of transmitting them. In Africa, several *Anopheles* species are suspected to play a role in the transmission of rodent *Plasmodium*, although few studies have formally identified these vectors in natural ecosystems (Makanga et al., 2016). Understanding the ecological associations between rodent hosts, parasites, and their vectors is essential to grasp the transmission dynamics, especially in the context of invasive species that may alter these interactions.

Thus, in Gabon, *Plasmodium* infections have been reported in multiple native rodent species, including *Lemniscomys striatus*, *Praomys* sp., and *Mastomys natalensis*, as well as in an invasive species, *Mus musculus domesticus* (Boundenga et al., 2019).

Despite the findings outlined above, there has been no prior evidence of natural *Plasmodium* infection in *R. rattus* (Boundenga et al., 2019). Given its shared habitat with several native rodent populations, *R. rattus* could potentially serve as a host for rodent *Plasmodium*. This possibility is supported by experimental studies showing that other *Rattus* species, such as laboratory *Rattus norvegicus*, are susceptible to *Plasmodium yoelii* and *P. berghei*, and can also be infected with other species like *P. vinckei* under immunocompromised conditions (Killick-Kendrick, 1978).

A recent survey of rodents conducted in rural villages of Gabon, where human settlements are in close proximity to natural ecosystems, confirmed the widespread presence of *R. rattus* (Mbou-Boutambe et al., 2024). This sampling effort provided a unique opportunity to investigate whether *R. rattus* has acquired rodent *Plasmodium* parasites. A total of 527 rodents was systematically screened using molecular biology techniques, with *R. rattus* accounting for approximately 90 % of the individual rodents tested. This study detected natural *Plasmodium* infections in *R. rattus* for the first time, providing novel insights into its role in malaria parasite ecology. The findings suggest that *R. rattus* is capable of harbouring *Plasmodium*, indicating that invasive rodents may play an underestimated role in rodent malaria ecology.

## 2 Materials and methods

### 2.1 Rodent trapping, identification and ethical statement

Rodent trapping campaigns were carried out following authorization by the Commission Scientifique d'Examen and the application for an Exploration Licence by Gabon (N◦AR005/20/MESRSTT/CENAREST/CG/CST/CSAR). The capture, handling, and euthanasia of animals, as well as the transfer of samples from Gabon to France, were carried out in accordance with the guidelines of the American Society of Mammalogists (Sikes and Care, 2016) and in strict compliance with the recommendations of the National Ethics Committee of Gabon (authorization N◦PROT/0020/2013I/S G/CNE).

**Fig. 1:**
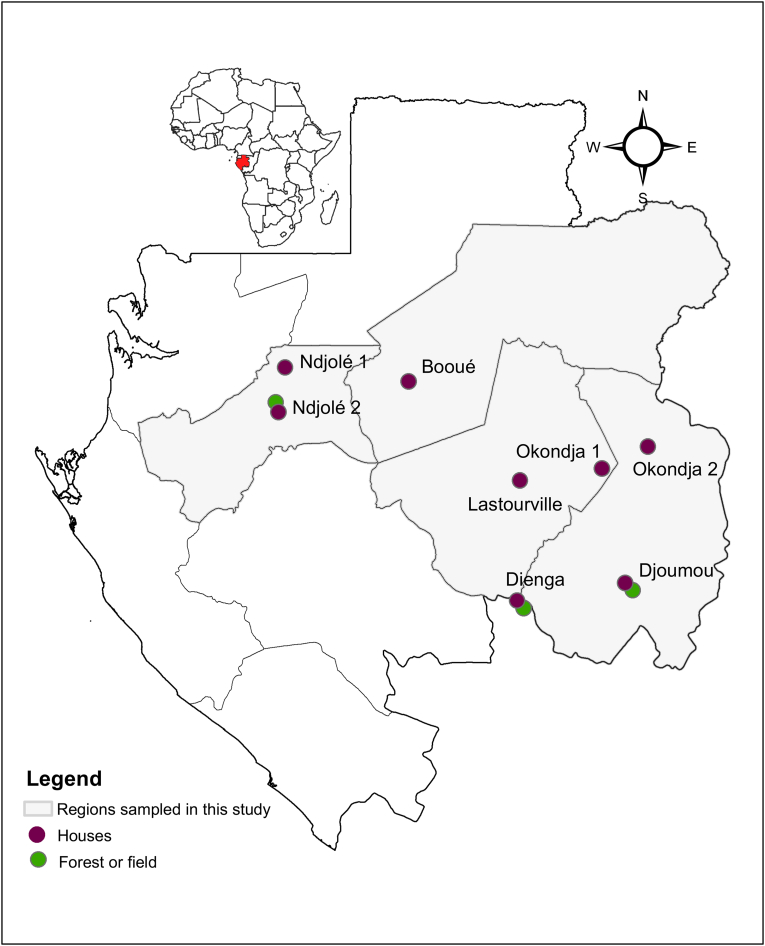
Location of rodent sampling sites in Gabon. Sites where only houses were sampled are denoted with a purple dot while sites where both houses and surroundings (forest or crop field) were sampled are denoted with both a purple and green dots.

Between October 2021 and June 2022, sampling of rodents was carried out in several rural areas in Gabon (Fig. 1) (Mbou-Boutambe et al., 2024). Inside each of these areas, several sampling sessions were performed in different villages. In all sites, the traps were set in houses for three consecutive nights. For three sites (Ndjolé, Dienga and Djoumou) additional sampling was also performed in the forest or in the crop fields close to the villages (see Table 1 for information on sampling effort per site). Tissues (brain, lung, intestine, liver) and blood were collected to investigate various pathogens circulating among the captured rodents Tissues (brain, lung, intestine, liver) were collected for the detection of pathogens circulating among small mammals. Samples were initially stored in liquid nitrogen and subsequently transferred to a −80 °C freezer at CIRMF for further molecular analyses. Approximately 50–200 μL of blood was collected from each individual, depending on the size of the animal, following procedures approved by the CENAREST ethics committee (Authorization No. AR0022/22/MESRSTT/CENAREST/CG/CST/CSAR) and in accordance with institutional animal welfare guidelines (Sikes and Care, 2016) . Only blood samples (collected from 527 individual rodents) were used for the detection of *Plasmodium* spp. in rodents. Samples were then stored in liquid nitrogen until storage in −20 °C freezer for further molecular analyses at the CIRMF.

**Table 1:** **Rodents species sampled in 11 sites in Gabon and observed number of *Plasmodium* infection per species and site.** For each site, an estimate of the trapping effort is provided. For each taxon, the number of infected individuals observed is provided as well as the number of individuals trapped. Exotic species: R. ra: *Rattus rattus* and M. mus: *Mus musculus domesticus*. Native species: H.un: *Hybomys univittatus*, H.ae: *Hylomuscus aeta*, L.st: *Lemniscomys striatus*, L.ro: *Lophuromys roseveari*, M. lo: *Malacomys longipes*, M.mi: *Mus minutoides*, O.hy: *Oenomys hypoxanthus*, P.ja: *Praomys jacksoni*, P.mi: *Praomys misonei* and P.pe: *Praomys petteri*.

Localities	Collection date	Trap nights	Total Captures	Rodent screenings													Total
				H.un	H.ae	L.st	L.nu	L.ro	M.lo	M.mi	M.mu	O.hy	P.ja	P.mi	P.pe	R.ra	
Booué_domestic	October 2021	1314	99	–	–	–	–	–	–	–	–	–	–	0/1	–	0/80	0/81
Dienga_domestic	February 2022	970	121	0/2	0/1	0/1	–	0/2	–	0/1	–	–	0/5	–	–	**2/84**	**2/97**
Dienga_forest	February 2022	176	31	0/5	–	0/8	–	0/9	0/2	–	–	0/2	–	–	0/1	–	0/28
Djoumou_field	June 2022	463	9	–	–	0/2	0/2	–	–	–	–	–	–	0/1	–	0/2	0/8
Djoumou_domestic	June 2022	291	14	–	–	–	–	–	–	–	–	–	–	–	–	0/14	0/14
Lastourville_domestic	April 2022	886	122	–	–	0/1	–	–	–	–	–	–	0/1	–	–	0/107	0/109
Ndjolé 1_domestic	June 2022	330	50	–	–	–	–	–	–	–	0/1	–	–	–	–	0/18	0/19
Ndjolé 2_domestic	June 2022	836	113	–	–	–	–	–	–	0/1	0/7	–	–	–	–	0/37	0/45
Ndjolé_forest	June 2022	178	2	–	–	–	–	–	–	–	–	–	–	–	–	–	–
Okondja 1_domestic	May 2022	588	130	–	–	–	–	–	–	–	–	**-**	–	–	–	0/94	0/94
Okondja 2_domestic	May 2022	126	52	–	–	–	–	–	–	–	–	–	–	–	–	0/35	0/35
Total		6158	743	0/7	0/1	0/12	0/2	0/11	0/2	0/2	0/8	0/2	0/6	0/2	0/1	**2/471**	**2/527**

Rodent species were identified by morphometric analyses following (Duplantier, 1982) and using genetic tools (barcoding using partial *cytochrome b* Sanger sequencing) (Nicolas et al., 2012) to avoid any ambiguity in the identification.

### 2.2 Molecular detection of parasites

Blood samples collected from captured rodents were screened for the presence of *Plasmodium* spp. For this purpose, DNA was extracted from approximately 200 μl of blood using the DNeasy Blood and Tissue kit (Qiagen, Courtabœuf, France) following the manufacturer's recommendations.

Total DNA was then used as a template for detecting the presence of malaria parasites through the amplification of a partial region of the *Plasmodium cytochrome b* gene as described in (Boundenga et al., 2016). PCR products (10 μL) were run on 1.5 % agarose gels in Tris-acetate-EDTA buffer to confirm successful amplification. Positive amplicons (∼868bp) were subsequently sequenced by Eurofins MWG (France).

To assign sequences to *Plasmodium* species, phylogenetic analyses were conducted using the cytochrome *b* sequences generated in this study (Genbank accession numbers PV590610 and PV590611) alongside a set of reference sequences obtained from Genbank (see Supplementary data for details). Sequences were aligned using the ClustalW algorithm implemented in the MEGA11 software package (Tamura et al., 2021). Phylogenetic relationships were inferred using the maximum likelihood method in PhyML available online at http://phylogeny.lirmm.fr/phylo_cgi/index.cgi (accessed on January 08, 2025). The GTR (General Time Reversible) + I (Invariable) model of nucleotide substitution was selected based on the Akaike Information Criterion as implemented in ModelTest (Dereeper et al., 2008). The robustness of the phylogenetic tree obtained was assessed using 100 bootstrap replicates.

## 3 Results

### 3.1 Rodent trapping

A total t of 6158 trap.nights resulted in the capture of 743 individual rodents (Table 1). Blood samples were successfully collected from 527 of these individuals, which were subsequently tested for the presence of *Plasmodium*. The rodents analysed belonged to thirteen species, with *R. rattus* representing the dominant one (471 individuals, 89.20 %) and *M. m. domesticus,* another invasive species, comprising 8 individuals (Table 1). The remaining individuals (n = 48) were of native species: *Hybomys univittatus* (N = 7), *Hylomyscus aeta* (N = 1), *Lemniscomys striatus* (N = 12), *Lophuromys nudicaudus* (N = 2), *Lophuromys roseveari* (N = 11), *Malacomys longipes* (N = 2), *Mus minutoides* (N = 2), *Oenomys hypoxanthus* (N = 2), *Praomys jacksoni* (N = 6), *Praomys misonei* (N = 2) and *Praomys petteri* (N = 1) (Table 1).

### 3.2 Detection of *plasmodium* parasites

**Fig. 2:**
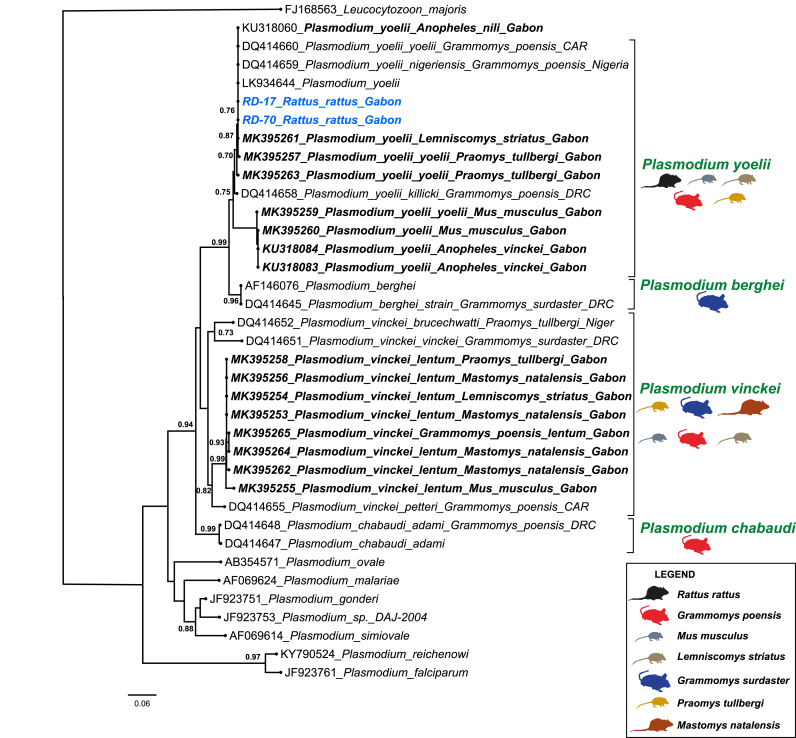
Phylogenetic relationships between the *cyt-b* sequences of *Plasmodium* parasites obtained in our study (in blue) and the sequences obtained from existing databases (in black). Names in bold represent samples obtained in Gabon by Boundenga et al. (2019). The tree was built using partial *cyt-b* sequences (868 bp-long). The boostrap values higher than 0.6 are displayed at the nodes. CAM: Cameroon and CAR: Central African Republic. For this analysis, the sequence of *Leucocytozoon majoris* (accession number FJ168563) was used as the outgroup.

The screening of the 527 rodent blood samples using nested PCR revealed two positive cases of *Plasmodium* infection (0.42 %), both found in *R. rattus* indivdiuals captured inside houses in the village of Dienga (Table 1, Fig. 2). No *Plasmodium* infection was detected in any of the 48 native rodent individuals from Gabon, nor in the other invasive species, *M. m*. *domesticus.*

### 3.3 Phylogenetic analysis

The sequences obtained from the two *Plasmodium* positive *R. rattus* samples were identified as *Plasmodium yoelii*, exhibiting 100 % nucleotide identity to previously reported subspecies *P. yoelii yoelii* and *P. yoelii nigeriensis*. The phylogenetic analyses placed these sequences within the clade of known *P. yoelii* isolates from rodents captured in Nigeria (Access number DQ414659) and the Central African Republic (access number DQ414660) (Fig. 2). Locally, the sequences showed complete homology with isolates of *P. yoelii* previously identified in *L. striatus* (accession number MK395261) and *Praomys tullbergi* (accession number MK395261), both captured in the Gabonese localities of Franceville and Bakoumba, respectively.

## 4 Discussion

This study provides the first molecular evidence of natural *P. yoelii* infection in *R. rattus* in Gabon, expanding the current understanding of the potential role of invasive rodents in malaria parasite transmission. While *R. rattus* is a known carrier of multiple zoonotic pathogens (Filippone et al., 2016, Houéménou et al., 2019, Mbou-Boutambe et al., 2024, Ramatla et al., 2022), its involvement in *Plasmodium* transmission has remained undectected. The detection of *P. yoelii* in *R. rattus* suggests that this species may acquire rodent malaria parasites in environments where it coexists with native rodent species, as previously observed in another invasive species, *M. m*. *domesticus* by Boundenga et al. (2019). This finding underscores the need to investigate host-switching events and transmission dynamics in areas undergoing ecological changes and species invasions.

Despite the detection of *Plasmodium* in *R. rattus*, the overall prevalence observed was very low (0.42 %) and observed cases were restricted to the village of Dienga, in the south east of Gabon. One potential explanation for the very low prevalence observed is limited exposure of *R. rattus* to competent vectors in the sampled habitats, as many *Plasmodium* parasites, including *P. yoelii*, rely on specific anopheline mosquito vectors for transmission (Makanga et al., 2016). The majority of *R. rattus* individuals in this study were captured in domestic and peri-domestic environments, where the density of sylvatic *Anopheles* species (such as *An. vinckei, An. nili,* and *An. marshallii*) may be lower than in forested areas (Longo-Pendy et al., 2022). Consequently, *R. rattus* individuals in peri-domestic settings may have reduced exposure to infected mosquitoes, leading to lower *Plasmodium* prevalence compared to native rodents in forest habitats (Lendongo Wombo et al., 2023) .

**The absence or low prevalence of *Plasmodium* infections in certain invasive rodent populations may also be explained by spatial heterogeneity in parasite distribution.** Previous studies have suggested that some regions may lack active transmission foci of rodent malaria parasites due to various ecological, environmental, or host community factors (Boundenga et al., 2019). In the case of Gabon, this dynamic may be further influenced by **ecological segregation between potential reservoir hosts and invasive rodents**. Native rodents such as *Grammomys* spp., suspected reservoirs of murine *Plasmodium*, are primarily **forest-dwelling (**Bryja et al., 2017**)**, whereas the black rat (*Rattus rattus*) is **strictly commensal**, occupying domestic and peri-domestic environments (Mangombi et al., 2016). Consequently, **opportunities for contact or spatial proximity** between these two groups are limited, thereby reducing the likelihood of **cross-species transmission**. This spatial patchiness in parasite occurrence may therefore lead to an **underestimation of the transmission potential** in invasive populations, when it in fact reflects the **localized absence of transmission (**Cottrell et al., 2012; Boundenga et al., 2019).

Finally, another factor contributing to the low infection rate observed in *R. rattus* may be host–parasite compatibility. Rodent malaria parasites, including *P. yoelii*, exhibit varying degrees of host specificity and are often closely associated with particular native hosts, such as *Grammomys poensis* in natural settings (Landau and Chabaud, 1994; but see Boundenga et al., 2016). Moreover, differences in susceptibility to rodent *Plasmodium* species have been documented even among native rodent hosts. In the case of the black rat, although experimental studies have shown that the laboratory rat (*Rattus norvegicus*), a closely related species, is a competent host for *P.* yoelii (Pattaradilokrat et al., 2022; Fu et al., 2012), to our knowledge, no experimental data are currently available regarding the susceptibility of *R. rattus*. It is therefore possible that *R. rattus* is a less competent host than other rodent species, which may contribute to its low observed infection rate in the wild, although both *R. rattus* and *R. norvegicus* are similarly competent hosts for a variety of diseases (Carter and Cordes, 1980; Himsworth et al., 2014; Ryll et al., 2017; Rothenburger et al., 2015).

It is noteworthy that no *Plasmodium* infections were detected in native rodents in this study, which contrasts with previous reports from Gabon that documented *Plasmodium* prevalence in *Grammomys poensis* (10 %), *Mastomys natalensis* (11.43 %), *Praomys* sp (10 %) and *Lemniscomys striatus* (3.33 %) (Boundenga et al., 2019). This discrepancy could be partly due to the relatively small sample size of native rodents in this study (n = 48), which may have limited the detection of infections.

This discrepancy also raises several ecological and epidemiological questions. How are *Rattus rattus* individuals acquiring infections in environments where native reservoirs appear to be uninfected? Could there be cryptic transmission pathways, such as low-level circulation in native hosts that went undetected due to limited sampling, or vector-borne intraspecific transmission within *R. rattus* populations? Further studies involving more comprehensive sampling of native rodent communities, along with entomological investigations, are needed to clarify these dynamics. However, it is important to note that species such as *Grammomys* sp., known reservoirs of *Plasmodium* in rodents (Boundenga et al., 2019), are notoriously difficult to capture, which may partly explain their underrepresentation in field surveys.

Despite the central role of rodent *Plasmodium* species in experimental research, particularly *P. yoelii* which has been cited in over 2500 publications since its first description in 1966 (although only 592 mention it explicitly in the title or abstract, according to Web of Science) these parasites have rarely been isolated from wild populations. Most laboratory strains currently in use are derived from a limited number of field isolates, the most recent of which were collected several decades ago and are maintained in the Edinburgh Rodent Malaria repository (latest isolates from 1967). The recovery of new field isolates would thus represent a significant advance, providing more ecologically and evolutionarily relevant models for the study of malaria biology, drug resistance, and parasite adaptation.

*R. rattus* is a known reservoir for other protozoan pathogens of public health importance, including *Toxoplasma gondii* (Hosseini et al., 2021) and *Leishmania* spp. (Pereira et al., 2017), further supporting its role as a competent host for a variety of zoonotic infections. This capacity highlights the potential of *R. rattus* to act as a bridge host for pathogens in disturbed habitats, especially those at the human–wildlife interface.

From an ecological perspective, the capacity of *R. rattus*, and more generally invasive species such as *M. m. domesticus* (Boundenga et al., 2019), to harbour *Plasmodium* parasites could have significant implications on parasite transmission dynamic, especially within or at proximity of human-modified environments.

## 5 Conclusion

This study presents the first evidence of *Plasmodium yoelii* infection in *R. rattus* in Africa, underscoring the need to reassess the role of invasive rodents in malaria ecology. While the infection prevalence was low, these findings suggest that *R. rattus* may integrate into local *Plasmodium* transmission cycles, warranting further investigation into its epidemiological significance. Future research should aim to elucidate the entomological context of these infections, particularly the identity and ecology of the mosquito vectors involved, as well as the host competence of *R. rattus* compared to native rodent species. Understanding the broader impact of invasive rodents on parasite transmission dynamics is especially relevant in landscapes undergoing rapid ecological change, such as deforestation, urban expansion, and agricultural intensification.

By advancing our knowledge of rodent-borne malaria transmission, including the capacity of invasive species to acquire and potentially spread *Plasmodium* parasites, we can better inform public health and conservation strategies. This includes anticipating emerging disease risks and mitigating the ecological consequences of biological invasions in both sylvatic and peri-domestic settings.

## CRediT authorship contribution statement

**Clark Mbou-Boutambe:** Writing – original draft, Visualization, Validation, Methodology, Investigation, Formal analysis, Data curation. **Larson Boundenga:** Writing – original draft, Visualization, Validation, Supervision, Resources, Project administration, Methodology, Investigation, Funding acquisition, Data curation, Conceptualization. **Fanny Degrugillier:** Investigation. **Philippe Gauthier:** Investigation. **Céline Arnathau:** Investigation. **Ana Rivero:** Writing – review & editing, Project administration. **Laurent Granjon:** Writing – review & editing, Project administration. **Virginie Rougeron:** Writing – review & editing, Validation, Supervision, Resources, Project administration, Methodology. **Franck Prugnolle:** Writing – review & editing, Visualization, Validation, Supervision, Resources, Project administration, Methodology, Funding acquisition, Data curation, Conceptualization.

## Funding sources

This study has been funded by ANR MICETRAL (Project number ANR-19-35CE-0010). We also thank the Gabonese Government and the National Research Agency (NRA) for their financial support. We are also grateful to people who consented the captures of rodents in their houses.

## Declaration of competing interest

The authors declare to have no conflict of interest with the study.

## Figures and Tables

**Table:** 

Access number	species	Host	Country
KU318060	*P.yoelii yoelii*	*Anopheles nili*	Gabon
DQ414660	*P.yoelii yoelii*	*Thamnomys rutilans*	Central African Republic
DQ414659	*P.yoelii nigeriensis*	*Thamnomys rutilans*	Nigeria
MK395261	*P.yoelii*	*Lemniscomys striatus*	Gabon
MK395257	*P.yoelii yoelii*	*Praomys tullbergi*	Gabon
MK395263	*P.yoelii yoelii*	*Praomys tullbergi*	Gabon
MK395259	*P.yoelii yoelii*	*Mus musculus domesticus*	Gabon
MK395260	*P.yoelii yoelii*	*Mus musculus domesticus*	Gabon
KU318084	*P. yoelii*	*Anopheles vinckei*	Gabon
KU8083	*P. yoelii*	*Anopheles vinckei*	Gabon
AF146076	*P. berghei*	NA	NA
DQ414645	*P. berghei strain*	*Grammomys surdaster*	RDC
DQ414652	*P.vinckei brucechwatti*	*Praomys tullbergi*	Nigeria
DQ414651	*P.vinckei vinckei*	*Grammomys surdaster*	RDC
MK395258	*P.vinckei lentum*	*Praomys tullbergi*	Gabon
MK395256	*P.vinckei lentum*	*Mastomys natalensis*	Gabon
MK395254	*P.vinckei lentum*	*Lemniscomys striatus*	Gabon
MK395253	*P.vinckei lentum*	*Mastomys natalensis*	Gabon
MK395265	*P.vinckei*	*Grammomys poensis*	Gabon
MK395264	*P.vinckei lentum*	*Mastomys natalensis*	Gabon
MK395262	*P.vinckei lentum*	*Mastomys natalensis*	Gabon
MK395255	*P.vinckei lentum*	*Mus musculus domesticus*	Gabon
DQ414655	*P.vinckei petteri*	*Thamnomys rutilans*	CAR
DQ414648	*P.chabaudi adami*	*Thamnomys rutilans*	DRC
DQ414647	*P. chabaudi adami*	*NA*	*NA*
AB354571	*P.ovale*	*NA*	*NA*
AF069624	*P.malaria*	*NA*	*NA*
JF923751	*P.gonderi*	*NA*	*NA*
JF923753	*Plasmodium* sp *DAJ-2004*	*NA*	*NA*
AF069614	*P.simiovale*	*NA*	*NA*
KY790524	*P.reichenowi*	*NA*	*NA*
JF923761	*P.falciparum*	*NA*	*NA*

## Appendix

Supplementary data *Plasmodium* sequences used as reference to construct the phylogenetic tree (see Fig. 2). For each sequence, the accession number, the host and the country where the parasite was collected is provided.
